# Reason and responsibility as a path toward ethical AI for (global) public health

**DOI:** 10.1038/s41746-025-01707-x

**Published:** 2025-06-03

**Authors:** Esther-Maria Antao, Aadil Rasheed, Anatol-Fiete Näher, Lothar H. Wieler

**Affiliations:** Hasso Plattner Institute for Digital Engineering, Digital Global Public Health, Digital Engineering, University of Potsdam, Rudolf-Breitscheid-Str. 187, D-14482 Potsdam, Germany

**Keywords:** Computational biology and bioinformatics, Environmental sciences, Health care, Scientific community, Social sciences

## Abstract

AI can enhance public health practice, but it requires careful consideration of ethical implications. We propose a reason-based framework to guide AI co-design and use for public health. AI systems must be developed with public health expertise, lived experience insights, and human accountability to ensure responsible outcomes. We advocate for ethical principles to be embedded throughout the AI lifecycle, thus proactively addressing risks while reinforcing trust in public health practice.

## AI in (Global) public health: driving innovation while ensuring ethical responsibility

(Global) Public health is an area of study, research, and practice that places a priority on improving health and achieving health equity for all people worldwide^1^. Public health practice differs essentially from healthcare in that populations and communities are at the heart of the profession rather than individual patients. With the rise of digital health technologies, artificial intelligence (AI) can play an increasingly prominent role in public health practice. Precision public health (PPH) is an emerging approach that aims to use advancements in digital technology and genomics to improve public health outcomes at the population level^2–4^. The World Health Organization (WHO) sees PPH as being about delivering “the right intervention at the right time, every time, to the right population”^5^. The integration of AI into public health systems could transform the landscape of the essential public health functions (EPHFs)^6^, including surveillance and monitoring, public health emergency management, disease prevention and health promotion, as well as community engagement, to name a few.

AI could indeed improve precision public health and enable more targeted, timely and effective public health interventions that better meet the unique needs of different populations. However, the rapid deployment of AI in public health raises critical ethical concerns and many challenges associated with AI use in public health and healthcare have been previously described^7,8^. Ethical frameworks are therefore essential to guide the responsible design and implementation of AI technologies for public health. From an ethical perspective, public health differs from clinical practice in key areas including Type of Interventions (Broader population-level actions versus individual medical interventions), Focus (Prevention and health promotion versus treatment of disease), Autonomy (Emphasis on relational autonomy, solidarity, and interdependence in public health versus individual autonomy in clinical ethics), Consent (Community consent and public engagement versus individual informed consent), Goals (promoting societal well-being while mitigating risks/harms towards a population versus prioritizing individual benefit in clinical care)^9^. This adds complexity to the ethical integration of AI in public health, as professionals are faced with a double dilemma when dealing with already existing complexities.

This perspective paper proposes a framework for AI co-design and use for public health, which focuses on the building blocks of human reasoning as an ethical and moral guidance. The framework is intended to enable public health practitioners to thoughtfully navigate the design and implementation of AI in public health, ensuring technology serves humans through empathy, cultural sensitivity, and a deep understanding of societal needs to achieve equitable and impactful outcomes. It is essential for public health professionals to foster ethical discussions and enable thoughtful, reflective, and logical ethical decision-making when integrating AI into essential public health functions.

## Working definitions

For the purpose of this article and in the context of public health we use the working definitions, some of which are adapted from the original reference as outlined in Table 1.

**Table 1:** Working definitions

Term	Definition
Knowledge	Public Health Information gathered through observation, data collection, curation and analysis by way of the scientific method, as well as that gathered through experience and practice that resemble insights not confined to the scientific method
Ethics	A set of moral principles or values to which public health professionals should adhere in order to justify possible courses of public health action^9^
Virtue	The quality of acting in a manner that shows high standards by abiding by a given set of moral principles defined for public health
Duty	Adhering to a set of rules with the main intention of not doing harm to or acting in the interest of a given community of population
Ethical challenges	Balancing individual freedoms with protecting a given community or population^9^
Ethical dilemma	Moral principles or values of public health professionals that are in conflict with values of other stakeholders^9^
Reason	The capacity to experience, learn, understand, judge and decide through logical and coherent thinking, in short, the sum of all intellectual powers
Human	A person with the ability to reason and act virtuously in duty through free choice
AI	A computational model that learns from a human to observe, understand and analyze information, as well as make decisions as a result of this understanding and analysis^21^

## Public health through the lens of knowledge and ethics

In line with the WHO definition of precision public health as “the right public health intervention reaching the right population at the right time and in the right way”, it is essential that public health professionals understand their dual role as hunters and gatherers of evidence-based information that informs public health interventions, and as communicators of this processed knowledge that actually leads to public health interventions, or to put it simply, the *hunter-gatherer* and the *ripple-maker*.

Knowledge in public health is a two-way street—it involves not only gathering and interpreting information (surveillance and monitoring) from data, communities and research, but also disseminating relevant evidence to the public, policymakers and stakeholders, after systematically processing and evaluating the available evidence (health promotion, policy advice and community engagement). Both phases, which form the essence of public health practice, are crucial for informed decision-making, timely interventions and the promotion of health equity. The performance of these tasks can sometimes lead to potential ethical dilemmas, for example, during public health emergency management (e.g., COVID-19 pandemic). Examples of these dilemmas include balancing honesty with the need to prevent panic in communication or weighing individual liberty against the protection of at-risk populations. Crisis communication is already a controversial ethical issue, due to the tension between individual liberty and the need for effective communication strategies^10^. The integration of AI to support these two phases has great potential but also raises several ethical concerns (Table 2). Public health practitioners already encounter numerous ethical challenges, requiring them to frequently navigate complex ethical questions by identifying ethical issues, articulating dilemmas, deliberating on options, and implementing solutions that remain open to revision, especially in rapidly evolving or uncertain situations. For example, acknowledging uncertainty (epistemic underdetermination) is recognized as a challenge in managing infodemics, posing a significant difficulty for public health^11^. Situation-based ethical analyses can help practitioners and organizations assess what they should do and why. The American Public Health Association offers guidance and suggests key ethical considerations which include permissibility, respect, reciprocity, effectiveness, responsible use of scarce resources, proportionality, accountability and transparency, and public participation^9^.

**Table 2:** Ethical concerns in potential use cases of AI integration in public health

Knowledge phase	Use case	Description	Ethical concerns
The Hunter-gatherer: Collecting and curating relevant public health information	Data-Driven Disease Prevention	Utilizing AI to enhance disease surveillance and prediction • Risk assessment • Contact tracing • Resource allocation	-Privacy and data protection issues -Inequity in data availability and digital access could further fuel the digital divide
The Ripple-maker: Disseminating relevant public health information	Health Education and Promotion	Utilizing AI to enhance public health communication • Principles from nudging to promote healthy behavior in population	-Loss of autonomy -Loss of freedom of choice -Bias in machine learning models and unequitable predictions

As AI begins to shape the future of (global) public health, it is crucial for AI designers and practitioners to adopt a human-centered approach. This means aligning AI tools with core values such as human dignity, justice, and autonomy^12,13^. Ensuring AI is both designed (together with public health experts) and implemented in an ethical manner might foster greater trust in the digital transformation of public health in the long run. When people recognize that public health professionals work in their best interest by respecting their rights and well-being, they are more likely to embrace and engage with AI-driven public health initiatives.

The idea of “knowledge” holds considerable relevance to (global) public health for two reasons: (1) Public health practitioners acquire (new) knowledge through evidence-based research and scientific inquiry, which is then processed through their own experience, understanding and judgment, (2) public health practice largely relies on the transfer of knowledge through communication or policy advice, which in turn relies on the recipient accepting this information through trust and belief in the facts presented^10,11^. Particularly, in the digital age, distinguishing between fact and fiction is challenging for the public, and accurately assessing information requires strong information literacy^11^. Knowledge is closely linked to reason, as it is through rational inquiry and critical thinking that we make sense of information, draw conclusions and build understanding.

Designing an AI system to augment or perform public health functions brings in a new dimension to traditional public health practice and will increasingly play an essential role in gathering, assessing, and even disseminating knowledge and information in the future. We propose a reason-based framework structured around the building blocks of human understanding - *Experience, Understanding, Judgment and Decision*^14^- for integrating AI ethically in public health practice (Table 3). For this proposition we assume that (1) The inherent nature of public health practice requires making decisions that impact entire communities or populations, creating ethical complexities that are further heightened by the intrinsic heterogeneity within these communities, (2) the role of ethical deliberations is crucial to ensure that public health actions align with moral principles and, (3) AI while being able to mimic stages of human reason remains limited in its capacity for genuine moral and ethical reasoning. We therefore assume that reason in this context is a uniquely human trait that AI cannot fully replicate in its depth and complexity. This complexity is shaped not only by logical reasoning, but also by human creativity and intuition - qualities that enable individuals to think abstractly, adapt to novel situations, and make ethical judgments beyond pre-defined rules. While AI can mimic patterns of reasoning, it lacks the spontaneity of creative insight and the nuanced intuition that guides human decision-making, particularly in uncertain or morally complex scenarios.

**Table 3:**
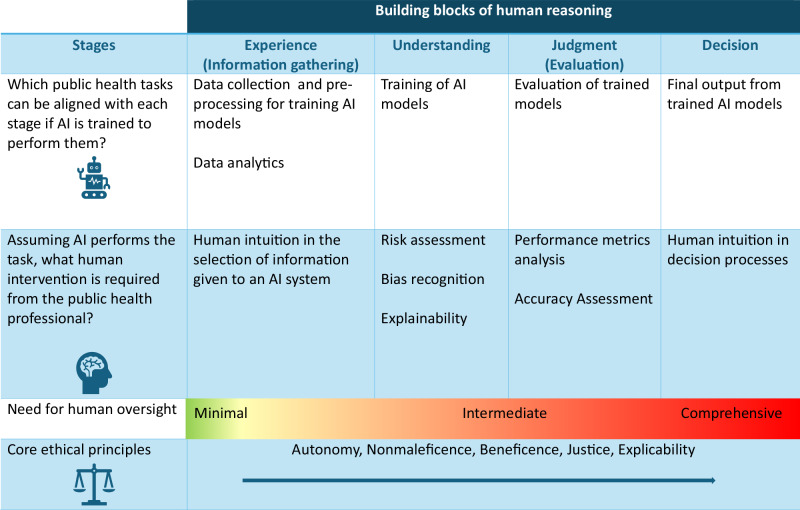
A reason-based framework for integrating AI in public health

AI systems could be developed to automate certain public health tasks^15^. Although AI is not yet widely integrated into public health functions, there are several areas where its use could be beneficial, with some examples briefly outlined, which include predictive analysis to forecast population health outcomes and disease outbreaks^16^, risk segmentation to cluster populations into different risk categories^17^ and leveraging natural language processing for public health communication^18^. In the future, AI could be ethically and effectively utilized to design health intervention nudges^19^. Furthermore, like AI-driven clinical decision support systems in healthcare^20^, AI could serve as a decision-support tool. While these tasks can be assigned to general-purpose AI or purpose-built systems, we argue that AI performing these functions must be designed with human oversight guided by principles of reasoning^21^. The development and deployment of such AI systems follow an inherently iterative process, while the reasoning behind the process unfolds in four distinct stages. The proposed framework advocates for varying levels of human intervention at each stage, ensuring that oversight remains integral throughout. After deployment, AI systems should undergo ongoing ethical oversight by humans.

From the ethical perspective, we begin by arguing both in an Aristotelian and Kantian sense^22–24^. For Aristotle, ethics requires both reason and virtue, where intellectual virtues (learned) differ from moral virtues (habits cultivated through practice). One of the main intellectual virtues for Aristotle is practical wisdom (*phronesis*), that is, a reasoned and true state of capacity to act with regard to human goods, or when of a person, the ability to deliberate well about what is good and expedient for oneself^22^. Moral reasoning, therefore, requires practical wisdom or what we widely term common sense, which is uniquely human. Guided by the *golden mean*—a balance between extremes—it reflects the essence of human nature. In context-specific situations, practical wisdom helps determine that golden mean, because it seeks not just a balance between extremes, rather can be applied intuitively depending on the situation at hand. Kantian ethics views morality as self-evident, rooted in the concept of *good will*—the intrinsic goodness of actions guided by a morally right will. Duty arises from respect for moral laws, and Kant’s *categorical imperative* requires acting out of moral obligation. Both Aristotle and Kant place reason at the center of ethical practice. However, while reason in an Aristotelian sense helps to cultivate virtues and balance extremes, Kant logically relies on reason to lay the foundation for developing universal moral laws and defining moral duty. Applying both Aristotelian and Kantian principles in the contexts described by this article, we argue that a reason-based framework could help cultivate virtuous action through habitual practice to ensure adherence to duty (moral principles). Public health professionals have a duty to make ethically guided decisions that promote population well-being. This involves balancing AI’s strengths and limitations (*golden mean*) to ensure that AI does not overstep human oversight, nor is its potential underutilized. In line with Kant’s categorical imperative, ethical AI integration must pass the test of universality, ensuring that its design and deployment in public health adhere to fundamental principles that public health professionals can broadly agree upon. As an ethical starting point, we suggest five principles, four of which were outlined by Beauchamp and Childress, which are intended to reflect universal values underlying rules of common morality^25^. These include (1) *respect for autonomy* (respecting the decision-making capacities of individuals), (2) *nonmaleficence* (avoiding the causation of harm), (3) *beneficence* (providing benefits and balancing these against risks and costs), and (4) *justice* (distributing benefits, risks and costs fairly)^25^. Floridi introduces a fifth principle for the ethical context of AI, *explicability*, which encompasses both intelligibility in an epistemological sense and accountability in an ethical sense^26^. However, modern ethical discussions are challenged by the normative justification of universal morality. Our framework centers on reason, drawing on human moral instincts and common sense to guide decisions on AI integration in public health. The five universal moral principles are intended to support decision-making, ensuring that moral maxims apply to everyone, regardless of personal views. The self-reflective, iterative cycle, that forms the basis of the framework, may be the key, in that through the stages of reasoning—experience, understanding, judgment and decision—the gap between normative and empirical justification can be narrowed, so that public health professionals can arrive at more objective criteria for identifying the benefits, harms and even general acceptability of the use of AI in public health. The focus is therefore not solely on conceptual universal rules and norms, but is based on a universal process that is inherent in all human reasoning^27^. It is beyond the scope of this article to go into details and ways of achieving objective criteria; however, the goal is to offer guidance, allowing for flexibility while maintaining moral standards and respecting the unique aspects of complex ethical situations.

Finally, transparent communication helps build trust between social beings^28^. However, the key messages that need to be delivered by the sender may not necessarily be understood and accepted by every recipient in the same manner^10,29^. A key lesson from the COVID-19 pandemic is the importance of transparent communication and consistent messaging from authorities^30^. When AI is used to perform essential public health functions, especially when it influences decision-making by both public health professionals and individuals, effective communication becomes paramount. Strengthening communication on AI in public health is essential for building trust, requiring ethical communication, community engagement, and equitable access to resources. Beyond AI’s practical benefits, this includes transparent discussions on risks and ethical dilemmas. By fostering open dialogue, public health authorities can promote informed decision-making and enhance trust in AI-driven health systems.

## Conclusion

While AI can enhance and augment the work and efforts of public health professionals, AI by itself cannot understand the lived human experience. Furthermore, AI as a ‘machine’ cannot be held accountable, nor can we expect the wider population to trust a ‘machine’. Therefore, AI systems need to be designed with input from public health experts before they are deployed. AI models should be trained with insights of lived human experiences, with the ultimate accountability for their performance resting with human public health practitioners.

In practical terms, human knowledge and moral reasoning are paramount when designing AI to perform essential public health functions. Ethical considerations must be at the forefront during the entire process of designing AI systems, rather than being considered only when an ethical dilemma arises out of the usage of such systems. A reason-based framework could offer guidance to practitioners when designing and using AI systems so that they can navigate complex ethical challenges from an early stage. Public health professionals must remain attentive and proactive, ensuring that every stage of planning and implementing AI is guided by principles that advance, rather than detract from, the achievement of public health goals.

## Data Availability

No datasets were generated or analyzed during the current study.
